# Brain/blood ratios of methadone and *ABCB1* polymorphisms in methadone-related deaths

**DOI:** 10.1007/s00414-021-02502-5

**Published:** 2021-01-17

**Authors:** S. Iwersen-Bergmann, S. Plattner, S. Hischke, A. Müller, H. Andresen-Streichert, H. Jungen, R. Erb, B. Beer-Sandner

**Affiliations:** 1 Department of Legal Medicine, University Medical Centre Hamburg-Eppendorf, Hamburg, Germany; 2 Institute of Legal Medicine, Innsbruck Medical University, Innsbruck, Austria; 3 Institute for Health Services Research in Dermatology and Nursing, University Medical Centre Hamburg-Eppendorf, Hamburg, Germany; 4 Institute of Legal Medicine, Faculty of Medicine, University of Cologne, Köln, Germany

**Keywords:** Methadone-related death, P-glycoprotein, *ABCB1* polymorphism, Brain/blood-ratio, rs1045642

## Abstract

Methadone is an opioid that often leads to fatalities. Interpretation of toxicological findings can be challenging if no further information about the case history is available. The aims of this study were (1) to determine whether brain/blood ratios can assist in the interpretation of methadone findings in fatalities; (2) to examine whether polymorphisms in the gene encoding the P-glycoprotein (also known as multidrug resistance protein 1 (MDR1) or ATP-binding cassette sub-family B member 1 (ABCB1)), which functions as a multispecific efflux pump in the blood–brain barrier, affect brain/blood ratios of methadone. Femoral venous blood and brain tissue (medulla oblongata and cerebellum) from 107 methadone-related deaths were analysed for methadone by gas chromatography-mass spectrometry. In addition, all the samples were genotyped for three common *ABCB1* single nucleotide polymorphisms (SNPs rs1045642, rs1128503, and rs2032582) using ion-pair reversed-phase high-performance liquid chromatography-electrospray ionization mass spectrometry (ICEMS). In nearly all cases, methadone concentrations were higher in the brain than in the blood. Inter-individual brain/blood ratios varied (0.6–11.6); the mean ratio was 2.85 (standard deviation 1.83, median 2.35). Moreover, significant differences in mean brain/blood ratios were detected among the synonymous genotypes of rs1045642 in *ABCB1* (*p* = 0.001). Cases with the T/T genotype had significantly higher brain/blood ratios than cases with the other genotypes (T/T vs. T/C difference (*d*) = 1.54, 95% CI [1.14, 2.05], *p* = 0.002; T/T vs. C/C *d* = 1.60, 95% CI [1.13, 2.29], *p* = 0.004). Our results suggest that the rs1045642 polymorphisms in *ABCB1* may affect methadone concentrations in the brain and its site of action and may be an additional factor influencing methadone toxicity.

## Introduction

Methadone is a μ-opioid receptor agonist and the primary drug used in maintenance treatment of heroin-dependent patients in Germany; however, it is also the most common cause of death in poisoning cases among drug addicts in Hamburg.

Methadone is usually administered as a racemic mixture of (R)- and (S)-methadone enantiomers by oral ingestion once daily with respect to its long half-life of 24–48 (− 55) h [1, 2]. (R)-methadone, better known as levomethadone, is also available for therapy. It is the active enantiomer at the μ-opioid receptor [3] and about twice as potent as the racemate*.*

Methadone is metabolized mainly in the liver by cytochrome P450 (CYP) enzymes. Among them, CYP2B6 and CYP3A4 are the main CYP enzymes with CYP2D6 and CYP2C19 making minor contributions [4, 5]. Polymorphisms in the genes that encode the CYP enzymes result in poor, extensive, or rapid metabolizers. Thus, a given dose of methadone can show broad inter-individual variations in methadone blood concentrations in living individuals. For example, Eap and coworkers [4] reported a 17-fold inter-individual variation of blood concentrations for the same dose of methadone. Furthermore, drug–drug interactions by co-medication have an impact on the pharmacokinetics of methadone [4–9]; in particular, antiretroviral drugs can affect methadone maintenance treatment (MMT) [9–13].

Therefore, successful treatment requires individual dose optimization. Several studies [14–17] have shown that overdose mortality is significantly higher within the first weeks of treatment, which poses a challenge for therapy and, in the case of death, makes discrimination of therapeutic and lethal methadone blood levels challenging. Therefore, additional tools that can help to clarify the cause of death in methadone-related cases are urgently required.

Opioid receptors, which are the sites of action of opioids, are located in the central nervous system; therefore, measuring methadone concentrations in the brain, rather than in the blood, may be more useful. Besides polymorphisms in CYP genes, polymorphisms in the *ABCB1* (ATP-binding cassette sub-family B member 1) gene also have been shown to influence the methadone doses required for effective MMT. Methadone is a substrate of P-glycoprotein transporter (P-gp), which is encoded by the highly polymorphic *ABCB1* gene [18]. P-gp is a multispecific efflux pump expressed by the endothelial cells of brain capillaries, also called the blood–brain barrier. Polymorphisms in *ABCB1* may potentially affect the concentrations of methadone in the brain. Intensively studied variants in the coding sequence of *ABCB1* are the synonymous single nucleotide polymorphisms (SNPs) 1236C>T (rs1128503) and 3435C>T (rs1045642) and the non-synonymous SNP 2677G>T (rs2032582). These three SNPs also are known to be in strong linkage disequilibrium, and the TT–TT–TT haplotype has been reported to minimize P-gp activity resulting in a fivefold higher probability of patients requiring a higher methadone dose compared with patients with other haplotypes [19]. The aim of this study was to investigate if brain/blood ratios of methadone correlate with these common *ABCB1* polymorphisms, which might reveal new insights into the toxicity of methadone.

## Materials and methods

### Materials

Racemic methadone (1 mg/mL in methanol) and methadone-D9 (100 μg/mL in methanol) were obtained from LGC (Wesel, Germany). Methanol was obtained from JT Baker (Deventer, Netherlands), and acetone, methylene chloride, dichloromethane, propanol-2, acetic acid, ammonia, and sodium dihydrogen phosphate were obtained from Merck (Darmstadt, Germany). All reagents were analytical reagent grade. Bond-Elut Certify cartridges were supplied by Agilent Technologies (Santa Clara, CA, USA).

### Experimental design

Autopsies were performed in the Department of Legal Medicine Hamburg, Germany, for legal reasons. In these cases, a full-scale toxicological investigation of all available specimens was performed using immunological, gas chromatography-mass spectrometry (GC-MS), and liquid chromatography (LC)-MS methods validated according to the guidelines of the Society of Toxicological and Forensic Chemistry [20]. Samples from medulla oblongata (hereafter referred to as medulla) and cerebellum were routinely taken if an opioid-related death was presumed. Samples were stored at − 20 °C for a maximum of 5–6 months until analysis. Results of samples from methadone positive autopsy cases were collected over a 5-year period.

### Instrumentation

The GC-MS analyses were performed on an Agilent Technologies (Waldbronn, Germany) 6890N gas chromatograph coupled to an Agilent Technologies 5975 mass-selective detector (MSD). A 30 m × 0.25 mm capillary column with a cross-linked (5% phenyl)-methylpolysiloxane coating of 0.25 μm was used, with splitless injection of 2 μL samples. The inlet pressure of the carrier gas (helium) was 0.772 bar, and the flow rate was set to 1 mL/min. The injection temperature was 250 °C. The oven was initially held at 100 °C for 4 min and then programmed to increase at 30 °C/min to 220 °C and held there for 9 min and then to increase at 50 °C/min to 300 °C and held there for 6 min. The MSD was used in the electron ionization (at 70 eV) selected ion monitoring mode to detect the target ion of methadone at 294 m/z and the qualifier ions at 72 m/z and 223 m/z and the target ion of methadone-D9 at 303 m/z and the qualifier ions at 78 m/z and 226 m/z.

### Analytical methods

Femoral venous blood and brain (medulla) of 107 methadone-related deaths were analysed for methadone by GC-MS. Cerebellum was available for 89 of the cases, so it also was analysed for methadone. It was not possible to distinguish between (R)- and (S)-methadone by the method used. We applied a well-established, validated and accredited GC/MS method, which has been used for routine methadone analysis in serum for more than 10 years. Validation parameters including accuracy, interferences, linearity of calibration, matrix effects, and in-process stability complied with the international recommendations [20, 21]. It was applied for the analysis of blood and brain with slight modifications.

The medulla and cerebellum specimens were homogenized using an Ultra Turrax device after addition of water (1:1 *w*/w). The homogenate of a drug-free medulla and drug-free blood were spiked with methadone and used as controls (QC) (QC1 0.1 mg and QC2 0.4 mg per kg brain or per L blood) and calibration standards of 0.01, 0.06, 0.1, 0.2, 0.3, 0.4, and 0.5 mg per kg brain or per L blood. To 0.5 g of each homogenate, 200 ng methadone-D9, 300 μL of a saturated potassium dihydrogen phosphate solution, and 1 mL acetone were added. The sample was vortexed for 2 min, followed by an ultrasonic bath (15 min) and centrifugation (10 min, 6000×g). The supernatant was transferred to a glass tube. Then, 1 mL acetone was added to the residue, and the procedure was repeated. The two supernatants were combined in the glass tube and evaporated at 40 °C. The dry residue was re-dissolved in 500 μL dipotassium hydrogen phosphate buffer (saturated dipotassium hydrogen phosphate solution and water 1:4 *v*/v, pH 9.5), and 1 mL water was added, and the pH was adjusted to pH 7.3–7.4, if necessary. A total of 1 mL of blood was diluted with water (1:1 v/v), and 200 ng methadone-D9 internal standard was added and vortexed (1 min). A total of 500 μL dipotassium hydrogen phosphate buffer (pH 9.5) and 200 μL of saturated potassium dihydrogen phosphate solution were added, and the pH was adjusted to pH 7.3–7.4, if necessary; vortexed for 1 min; and centrifuged (10 min, 6000×g).

The preparations from medulla, cerebellum, and calibration and control standards or blood were extracted by solid-phase extraction using a Bond-Elut Certify cartridge (3 mL) that had been preconditioned with 3 mL propanol-2, 3 mL methanol, and 3 mL water. After passage of the sample, the cartridge was washed with 2 mL water and 1 mL acetic acid (0.1 mol/L). The column was dried for 1 min, washed with 2 mL methanol, and dried for 3 min under vacuum. Elution was performed with 1.2 mL of a mixture of methylene chloride/propanol-2/ammonia (78:20:2 *v*/v). The extract was dried under a gentle stream of nitrogen and reconstituted by the addition of 60 μL methanol. Two microliters were injected into the GC-MS system.

The calibration curves in blood and brain (including seven spiked calibration concentrations 0.01 mg/kg (mg/L) to 0.50 mg/kg (mg/L) fitted to a linear regression function. Calibration derived from blood and brain standards was in line with the routinely used calibration from serum calibrators. The limit of quantification (LOQ) in blood (medulla) was 0.01 mg/L (0.01 mg/kg), and the limit of detection (LOD) was 0.002 mg/L (0.006 mg/kg). The analytical recovery of methadone was determined at two different concentrations in blood (medulla) and yielded 64.4% (63.9%) for QC1 and 68.4% (66.2%) for QC2. Accuracy was assessed at QC concentration levels on different days. Intraday coefficient of variation in blood (medulla) was 5% (5%) for QC1 and 4.1% (4.4%) for QC2. Interday coefficient of variation was 7.6% (8.2%) and 6.8% (6.2%) respectively. Bias was < 5%. Regular external quality control was performed by periodical proficiency testing in serum.

### Genotyping by polymerase chain reaction ion-pair reversed-phase high-performance liquid chromatography-electrospray ionization mass spectrometry

Blood samples were genotyped for three common *ABCB1* polymorphisms (rs1045642, rs1128503, rs2032582) by PCR-ICEMS [22, 23]. The study was approved by the local ethics committee. Total genomic DNA was extracted from collected blood samples using a Chelex extraction protocol [24]. The DNA content was determined by spectrophotometry (ND-1000, Nano-Drop Technologies, Wilmington, DE). The PCR mixtures contained 1× Advantage 2 SA PCR buffer (Takara Clontech, Mountain View, CA), 312.5 μM of each dNTP, 350 nM forward, and 350 nM reverse primers (Table 1) and 1× Advantage 2 Polymerase Mix (Takara Clontech), 2.5 μl DNA extract, and H_2_O to 20 μl. Amplifications were performed in 96-well polypropylene plates on a GeneAmp PCR System 9700 thermal cycler (Thermo Fisher Scientific, Waltham, MA, USA) using the standard thermal ramp-speed setting. The thermal cycler protocol was initial template denaturation/polymerase activation at 95 °C for 1 min, followed by 40 cycles of denaturation at 95 °C for 15 s, primer annealing at 62 °C for 30 s, and primer extension at 72 °C for 30 s, with the final extension for 7 min.

**Table 1:** Primer sequences

SNP	Forward primer	Reverse primer
rs1045642	AGCACACCTGGGCATCGT	CAGTGACTCGATGAAGGCATGT
rs1128503	TGTGTCTGTGAATTGCCTTGAA	CACAGCCACTGTTTCCAACC
rs2032582	CCATCATTGCAATAGCAGGAGT	GCAGTAGGGAGTAACAAAATAACACTG

An ultimate fully integrated capillary HPLC system (LC-Packings, Amsterdam, The Netherlands) was used for chromatographic separations. Sample injection was performed by a Famos micro-autosampler (LC-Packings) equipped with a 2-μl loop. The flow rate was set to 3 μL. The 50 × 0.2 mm i.d. monolithic capillary column was prepared according to the published protocol [25]. Denaturation of the amplicons was enabled by setting the column temperature to 70 °C. Separation of single-stranded DNA molecules was accomplished with a 7-min gradient of 5–80% acetonitrile in 25 mM cyclohexyldimethylammonium acetate. Eluting nucleic acids were detected online by electrospray ionization (ESI)-MS on a QSTAR XL mass spectrometer (Sciex, Framingham, MA, USA) equipped with a modified TurboIonSpray source [25]. Mass calibration and optimization of instrumental parameters were performed in the negative ion mode as described previously [26, 27]. The spray voltage was set to − 4 kV. Gas flows of 15 arbitrary units (nebulizer gas) and 30 arbitrary units (turbo gas) were employed. The temperature of the turbo gas was adjusted to 200 °C. The accumulation time was set to 1 s. Mass spectra were recorded between 800 and 1200 m/z on a personal computer operating with the Analyst QS software (version 1.0, service pack 8 and Bioanalyst extension, Sciex). The measured molecular masses were converted to genotype information by matching them to allele-specific masses.

### Statistical analysis

Methadone concentrations of brain and blood were reported as mean values, standard deviation (SD), median, and minimum and maximum, and genotypes were reported as frequencies.

For statistical analyses, the ratios of the medulla/blood and cerebellum/blood methadone concentrations were calculated. Differences in mean were examined using the *t*-test for paired and unpaired samples. One-way analysis of variance was used to analyse the difference between the mean methadone ratio and the different alleles of the three SNPs, rs1045642, rs1128503, and rs2032582. A post hoc test with Bonferroni correction was used to determine the mean differences between the alleles. Logarithmic transformation (natural logarithm, ln) of methadone ratio was done beforehand in order to approximate normal distribution. Distribution was evaluated graphically since a preliminary test on normality would increases the type one error. We preferred the one-way analysis of variance with transformed data over non-parametric statistics since parametric tests usually have more power. In order to interpret the results of the one-way analysis, we transformed back the estimates with the reverse function of the natural logarithm: the exponential function (exp). *P* values <0.05 were considered statistically significant. All statistical analyses were performed with IBM SPSS Statistics for Windows, version 26.0.

## Results

### Samples

The brain and blood samples included in this study were from individuals whose deaths were methadone-related (79 male, 28 female; average age, 41 years (21–65 years)). There was no evidence of any other causes of death from macroscopic or microscopic findings. In 19 cases (17.7%), death was attributable to methadone toxicity alone, whereas for the remaining 82.3% of cases, death was attributed to a combination of methadone and blood alcohol levels > 0.3% (19.2%), benzodiazepines (31.0%), opiates/heroin (17.2%), or cocaine (13.9%). The case histories indicated that 30 individuals (28.0%) had not taken part in an MMT programme (non-MMT) and that 24 individuals (22.4%) had been in MMT at the point of death. No reliable information was available for the remaining 53 cases. In all three groups MMT, non-MMT, and unknown, some deaths showed evidence of intravenous use of (diverted) methadone. Femoral venous blood and medulla samples were analysed for methadone concentrations by GC-MS. The results are shown in Table 2. The difference between the mean peripheral blood concentrations in the MMT and non-MMT groups was statistically significant (*p* < 0.05).

**Table 2:** Methadone venous blood, medulla oblongata, and cerebellum concentration in different collections of methadone-related deaths with subdivision in two treatment groups. Differences between the mean venous blood concentrations of methadone maintenance treatment (MMT) and non-MMT (*p* < 0.05).

	*N*	Methadone (mg/L or mg/kg) Mean ± SD	Methadone (mg/L or mg/kg) median	Range mg/L or mg/kg (lowest to highest)
All cases venous blood	107	0.74 ± 1.19	0.47	0.06–11.5
All cases medulla oblongata	107	1.66 ± 2.20	1.20	0.18–20.8
All cases cerebellum	89	1.63 ± 2.49	1.14	0.4–22.9
Non-MMT venous blood	30	0.47 ± 0.36	0.36	0.06–1.08
Non-MMT medulla oblongata	30	1.18 ± 0.95	0.71	0.31–2.68
MMT venous blood	24	0.98 ± 0.77	0.74	0.15–2.5
MMT Medulla oblongata	24	2.11 ± 1.20	2.2	0.4–4.0

### Brain/blood ratios

The mean methadone concentrations in medulla and cerebellum were 1.66 mg/kg (SD 2.21) and 1.63 mg/kg (SD 2.48), respectively (Table 2). The mean medulla/blood ratio was 2.85 (SD 1.83) with a range of 0.71–11.67 (Table 3). The mean cerebellum/blood ratio was 2.85 (SD 2.08). The difference between the mean medulla/blood and mean cerebellum/blood ratios was not statistically significant (*p* = 0.3).

**Table 3:** Methadone medulla/blood ratios in different collections of methadone intoxications with subdivision in the two treatment groups; methadone maintenance treatment (MMT) and non-MMT

	*N*	Medulla/blood ratio*: mean	Medulla/blood ratio*: median	Medulla/blood ratio*(lowest to highest)
All cases	107	2.85	2.35	0.71–11.67
MMT	24	2.78	2.43	1.1–5.9
Non-MMT	30	3.14	2.50	0.6–11.67

*The unit of the brain/blood ratio is kg/L

### Genotyping for three common ABCB1 polymorphisms

**Fig. 1:**
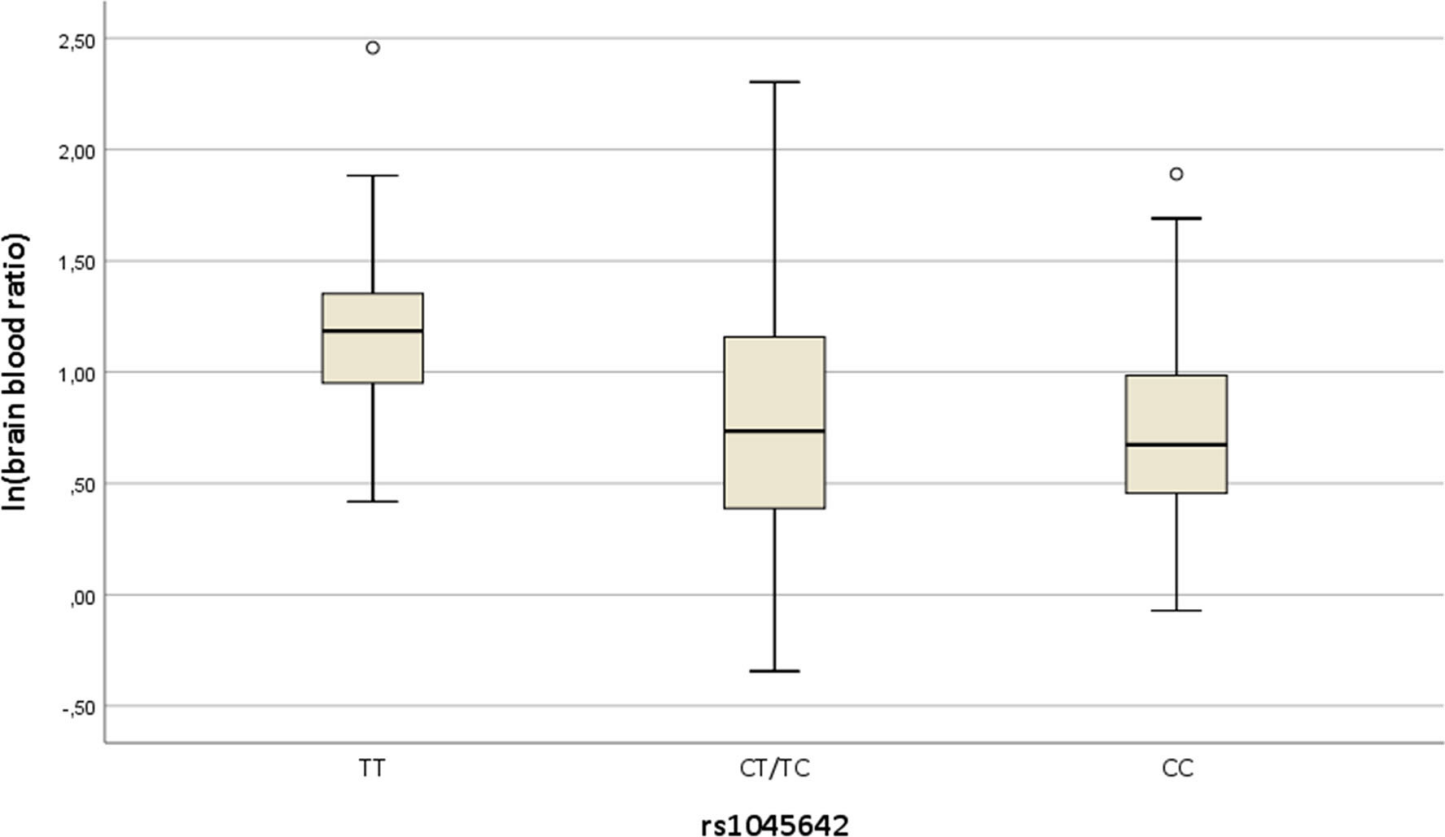
Box plot of logarithmic brain/blood ratio of different alleles of SNP rs1045642

All the samples were genotyped for three common *ABCB1* SNPs (rs1045642, rs1128503, rs2032582). The distribution of the genotypes in the three SNPs is shown in Table 4. The T/T genotype of rs1045642 was equally distributed between the MMT and non-MMTgroup. Significant differences in mean medulla/blood ratios were detected between the different genotypes (*p* = 0.001). The distribution of the genotype-specific medulla/blood ratio of the different alleles of SNP rs1045642 are shown Fig. 1. For SNP rs1045642, the medulla/blood ratios of the T/T genotype were significantly higher than those of the other genotypes (T/T vs. T/C distance (*d*) = 1.54, 95% CI [1.14, 2.05], *p* = 0.002; T/T vs. C/C *d* = 1.60, 95% CI [1.13, 2.29], *p* = 0.004) (Tables 5 and 6).

**Table 4:** Distribution of the different alleles in the 3 different SNPs

		Frequency	Percentage (%)
rs1045642	Total	107	100.0
	TT	28	26.2
	CT/TC	55	51.4
	CC	24	22.4
rs1128503	TT	17	15.9
	CT/TC	52	48.6
	CC	38	35.5
rs2032582	TT	21	19.6
	GT/TG	56	52.3
	GG	27	25.2
	AG	3	2.8

**Table 5:** SNP rs1045642—mean logarithmic and transformed back (with exponential function) medulla/blood ratio values with corresponding 95% confidence intervals

rs1045642	N	Mean (SD) ln	Exp (Mean (SD)): transformed back (exponential)	95% confidence interval: ln	95% confidence interval: transformed back (exponential)
TT	28	1.21 (0.41)	3.36 (1.50)	[1.05, 1.37]	[2.87, 3.93]
CT/TC	54	0.78 (0.58)	2.19 (1.79)	[0.63, 0.94]	[1.87, 2.57]
CC	24	0.74 (0.48)	2.09 (1.61)	[0.54, 0.94]	[1.71, 2.56]
Total	106	0.89 (0.55)	2.43 (1.74)	[0.78, 0.99]	[2.18, 2.70]

**Table 6:** Post hoc test: mean difference of methadone medulla/blood ratio with corresponding 95% confidence intervals and *p* values after Bonferroni correction

rs1045642		*d* mean difference (I–J)		95% confidence interval		*p*-value
(I)	(J)	ln	Transformed back (exponential)	ln	Transformed back (exponential)	
TT	CT/TC	0.43	1.54	[0.13, 0.72]	[1.14, 2.05]	0.0019
	CC	0.47	1.60	[0.12, 0.83]	[1.13, 2.29]	0.0042
CT/TC	TT	− 0.43	0.65	[− 0.72, −0.13]	[0.49, 0.88]	0.0019
	CC	0.05	1.05	[− 0.26, 0.36]	[0.77, 1.43]	1
CC	TT	− 0.47	0.63	[− 0.83, 0.12]	[0.44, 1.13]	0.0042
	CT/TC	− 0.05	0.95	[− 0.36, 0.26]	[0.70, 1.30]	1

No significant differences in the distribution of genotype-specific medulla/blood ratios of the different alleles were detected for SNPs rs1128503 (*p* = 0.5) and rs2032582 (*p* = 0.12).

## Discussion

Postmortem methadone blood levels are difficult to assess because of high inter-individual variability of pharmacokinetic, pharmacodynamic, and opioid tolerance levels. Furthermore, blood levels do not necessarily reflect the methadone concentration at its site of action, the brain. We consider that the concentration of methadone in the brain reflects the effective concentration in the central nervous system with regard to the fatal effects of respiratory depression. Therefore, we determined the concentration of methadone in two parts of the brain in methadone-related fatalities (medulla and cerebellum).

Medulla was chosen for methadone analysis because it has a high density of μ-opiate receptors [28]. As a reference, we also analysed the methadone concentration in the cerebellum. Other studies have analysed methadone in the frontal lobe [29], grey matter of the frontal lobe [30], or simply “brain” [31].

Although the medulla has a higher density of opioid receptors than the cerebellum, we found that the concentrations of methadone were very similar in both parts of the brain (Table 2). Therefore, we refer only to medulla in the discussion that follows.

The methadone concentrations in blood and medulla were significantly higher in the individuals who were participating in an MMT programme at the point of death. These results are in line with previous studies of methadone-related deaths [31–33] where the MMT group had significantly higher mean blood methadone concentrations than the non-MMT group. The differences between the two groups can be explained by the regular daily intake of methadone, resulting in a higher tolerance level towards methadone in the MMT group. Therefore, only considerably higher methadone concentrations led to death by respiratory depression of individuals in the MMT group. Moreover, the higher blood levels of MMT patients might be explained by the accumulation of (R)-methadone when taken regularly [31]. (R)-methadone has a longer half-life (mean 38 h) than (S)-methadone (mean 29 h) [4]. It seems reasonable to assume that the higher blood concentration of methadone in the MMT group was responsible for higher concentration detected in the brain in the MMT group.

The blood–brain barrier is formed by a tight junction of brain capillary endothelial cells that must be passed by drugs before they enter the brain parenchyma. Lipophilic drugs like methadone can cross the blood–brain barrier by diffusion. Our results show that, in nearly all the cases, methadone concentrations were higher in medulla than in blood (mean medulla/blood ratio 2.85), which corresponds very well with the results of Holm and Linnet [29] who reported a total methadone mean frontal lobe/blood ratio of 2.6 (1.3–3.9) in six methadone-related deaths. In a later study [30], the same authors reported a mean frontal lobe/femoral blood ratio of 2.3 (0.49–21.7; *N* = 105) for (R)-methadone. Liu and coworkers [34] found a brain/blood ratio for methadone of 2.7 in rats. We found considerable inter-individual variation of the medulla/blood ratios (0.6–11.6), which is similar to that reported by Holm and Linnet [30]. The smaller inter-individual variation (1.3–3.9) found in the first study of Holm and Linnet [29] can be explained by the small number of examined cases (*N* = 6) compared with the numbers in their second study (*N* = 105) and in our study (*N* = 107).

Our results suggest that differences in methadone transport out of the brain and into the blood may explain the large variations in brain/blood ratios.

P-gp acts as a multispecific efflux pump transporting various endogenous compounds as well as drugs from intracellular to extracellular domains [35]. P-gp is found in intestinal, kidney, and hepatic cells, as well as in the endothelial cells of brain capillaries (blood–brain barrier).

The literature does not provide consistent results regarding the question whether methadone is a substrate for P-gp in the blood-brain barrier.

In vitro and animal studies suggest that methadone is a substrate for the efflux transporter P-glycoprotein. Crettol et al. [18] showed that methadone was a substrate of P-gp in *ABCB1*-transfected cells, and only weak stereoselectivity in methadone transport was observed towards the (S)-enantiomer. The authors postulate that P-gp may affect the pharmacokinetics and pharmacodynamics of methadone [18]. Gibbs and coworkers [36] concluded from their in vitro results on P-gp-mediated ATP hydrolysis that methadone had a single binding site on P-gp unlike loperamide, which had two binding sites. These authors suggested that the two binding sites explained the fourfold higher P-gp-mediated opioid transport rate of loperamide compared with that of methadone.

Hassan et al. [37] investigated the P-gp affinity status in vivo and in vitro with four assays in mdr1a/b (+/+) versus mdr1a/b (−/−) mice. Methadone was found to be positive in all four assays. The authors state a strong evidence and methadone appearing to be a P-gp substrate. Wang et al. [38] studied methadone concentrations in blood and brain of *Abcb1a*^*−/−*^ knockout mice compared with those of mice with the *Abcb1a*^*+/+*^ wild type. The blood concentrations were found to be similar in both groups after intraperitoneal methadone application, whereas the brain concentrations in the *Abcb1a*^*−/−*^ knockout mice were markedly higher than in the wild-type mice (23- and 15-fold, respectively). In human, no loss-of-function mutation in P-gp has been discovered so far [39]. The T-variant of SNP rs1045642 has been associated with impaired function and/or abundance of P-gp in vitro and in vivo. [40]. Because P-gp effluxes compounds from the brain into the bloodstream, it has been hypothesized that impaired P-gp functionality may lead to increased accumulation of particular endogenous compounds in the brain [41].

Some clinical studies showed that antiretroviral agents (ritonavir and efavirenz) [42, 43] affect the concentration-response relationship of methadone in plasma (determined from the decrease in pupil diameter by opioids). However, two further clinical studies found no role for quinidine-inhibitable or cyclosporine-inhibitable transporters in methadone brain access [44, 45] suggesting that P-gp is not the principal determinant of methadone brain access in humans [46]. These findings are consistent with subsequent studies using cells which over-express specific transporters found that methadone was not a substrate for uptake or efflux transporters [47].

Our findings suggest that the *ABCB1* genotype may have a significant impact on the methadone brain/blood ratio, as we found out that the homozygous TT allele of SNP rs1045642 was associated with a significantly higher medulla/blood ratio of methadone compared with the ratios for the other genotypes. Therefore, individuals with the TT genotype had much higher methadone concentrations in the brain compared with the concentrations in blood and may have higher risk of dying at lower blood methadone concentrations than individuals with the other genotypes. These results suggest that carriers of the TT variant of rs1045642, who are likely to have higher methadone concentrations in the brain than assumed from their blood concentrations, might need lower doses of methadone for successful treatment.

A few further studies have investigated the association between methadone doses in MMT and SNP rs1045642. Zahari and coworkers found out that patients undergoing MMT with diplotype CGC/TTT (1236C>T (dbSNP rs1128503), 2677G>T/A (dbSNP rs2032582), and 3435C>T (dbSNP rs1045642) had 32.9% higher dose-adjusted serum methadone concentration over the 24-h dosing interval [48]. Levran et al. [19] investigated *ABCB1* multilocus genotype patterns, including in SNPs (rs1045642–rs2032582–rs1128503), in 98 MMT patients and found that the TT–TT–TT and TT–GT–CT patterns, both with the homozygous TT genotype of rs1045642, were linked to the need to administer high methadone doses (> 150 mg/day) for treatment stabilization. Hung et al. [49] and Coller and colleagues [50] reported similar results, whereas Crettol et al. [51] found only a small influence, and Fonseca [52] found no influence of the rs1045642 genotype on the required dose of methadone. Dennis and coworkers [53] performed a meta-analysis of the pooled results of *ABCB1* SNP studies that examined the association between rs1045642 genotypes and methadone dose. They concluded that methadone dose was not significantly associated with *ABCB1* polymorphisms. Barrat and coworkers [54] compared *ABCB1* haplotypes (with and without controlling for the μ-opioid receptor (*OPRM1*) genotype) and postulated that additional environmental or genetic factors may confound *ABCB1* pharmacogenetics in different MMT settings. They identified two interacting pharmacogenetic determinants of the MMT response: *ABCB1*, where variants were associated with lower methadone requirements, and *OPRM1*, where the variant was associated with higher methadone requirements.

The discrepancy arising from published data shows the complexity of the pharmacodynamics and pharmacogenetics of methadone. This study has some limitations because we did not include other polymorphisms, such as the relevant CYP enzymes and μ-opioid receptors. Even so, our findings suggest that the *ABCB1* genotype may have a significant impact on the methadone brain/blood ratio. To our knowledge, this is the first time that *ABCB1* polymorphisms have been shown to affect the brain/blood ratios of methadone in human. Although this finding cannot be interpreted independently from other genetic variants, the results of this study could be considered as a basis for further investigations of the influence of ABCB1 polymorphisms on methadone toxicity in fatal overdose cases.

In the present study, information on whether the deceased had participated in an MMT programme was missing in about half of the cases. Considering that the genotype of SNP 3435C>T (rs1045642) was equally distributed between the MMT and non-MMT groups, this limitation does not seem to be decisive.

One further limitation of this investigation is that the analysis for methadone was not done enantioselective. However, at the time of conducting this study, no enantioselective method was available, and the stereoselectivity of methadone transport by P-gp was shown to be weak in vitro [18].

## Conclusions

Tolerance towards opiates, co-consumption of other drugs, and the route of administration (oral/intravenous) influence the toxicity of methadone. Our results suggest that measuring the brain concentration of methadone could support toxicological interpretations and that polymorphisms in *ABCB1* may influence methadone toxicity.
